# Tuberculosis among foreign-born populations in the Western Pacific Region: emerging trends and analysis from 2008 to 2023

**DOI:** 10.1186/s41182-025-00866-7

**Published:** 2025-12-19

**Authors:** Francisca S. Y. Wong, Fukushi Morishita, Kyung Hyun Oh, Huong Thi Giang Tran, Rajendra-Prasad Yadav

**Affiliations:** World Health Organization Regional Office for the Western Pacific, Manila, Philippines

**Keywords:** Tuberculosis, Migration, Epidemiology, Foreign-born, Western Pacific Region

## Abstract

**Background:**

Migration significantly influences tuberculosis (TB) epidemiology in the Western Pacific Region (WPR), posing challenges to its control and elimination. This study examines the burden of TB among foreign-born individuals at regional and national levels in the WPR.

**Methods:**

Using data from the WHO Global TB Database and the United Nations’ International Migrant Stock dataset, we analysed the number and proportion of foreign-born TB case notifications across the region from 2008 to 2023. We also compared estimated TB incidence among international migrants with regional and national averages in WPR destinations.

**Results:**

Foreign-born TB notifications increased from 5,639 in 2008 to 10,056 in 2023, with trends varying across the WPR. Malaysia (40.4%), Japan (16.0%), and Australia (12.7%) accounted for the largest caseloads in 2023. Between 2021 and 2023, foreign-born TB cases represented 0.8% of total case notifications in the region, with Australia (89.9%), and New Zealand (86.5%) reporting the highest proportions. As of 2020, international migrants in the WPR (24.8 million, 77.9% of whom originated from high-burden countries) had an estimated TB incidence rate of 130 per 100,000, exceeding national averages in many countries and areas.

**Conclusions:**

Significant disparities remain in the foreign-born TB burden across the WPR. Strengthening surveillance, improving data comparability, and enhancing cross-border collaboration through migrant-sensitive approaches may help address existing gaps and support progress towards the End TB targets.

## Background

Tuberculosis (TB) is a leading cause of morbidity and mortality worldwide, with an estimated 10.8 million new cases and 1.3 million deaths in 2023 [1]. According to the WHO Global Tuberculosis Report 2024, the Western Pacific Region (WPR) accounted for approximately 1.8 million new TB cases in 2023, representing 17% of the global disease burden. Countries and areas in the region vary widely in population size, income level, stage of development, and TB epidemiology. While several countries have achieved notable reductions in TB incidence over the past decade, the Philippines continues to have one of the highest TB incidence rates globally (> 500 per 100,000 population). Papua New Guinea (> 400 per 100,000) and Viet Nam (180 per 100,000) also bear substantial disease burdens [1].

Migration, both internal and external, poses significant challenges to TB control and progress towards achieving the End TB targets [2, 3]. Globalisation has intensified population movements, raising emerging public health concerns, including the distribution and control of TB [4]. Migrating populations are thought to carry a hidden portion of the TB burden, complicating diagnosis, treatment continuity, and long-term disease management [1, 5]. Internal migration can fragment care and weaken linkage to health systems, while external migration may create reservoirs of undiagnosed or untreated TB across borders. Evidence from high-income settings has demonstrated that international migration can reshape TB epidemiology and complicate existing control strategies, underscoring the need for tailored interventions [5].

In the WPR, key drivers of migration include economic and employment opportunities, educational pursuits, and increasingly, climate- and environment-related displacement within and between countries [2, 6, 7]. For instance, lower-income countries may serve as sources of labour migrants to higher-income countries within the region, while Pacific Island Countries face growing displacement pressures due to climate change [2, 4]. These complex and dynamic migration patterns present major challenges for TB surveillance, case detection, and care continuity [1]. Despite the scale of these movements and their potential impact on the TB burden, analyses quantifying foreign-born TB in the WPR remain limited, leaving critical gaps in the evidence base. Here, we examine trends in foreign-born TB case notifications in the region, and compare the estimated TB incidence among international migrants with regional and national averages to improve understanding and support more targeted public health interventions.

## Methods

### Data source

Data on annual national TB notifications and estimated incidence rates were obtained from the WHO Global Tuberculosis Database [8]. Key indicators included total cases notified, cases that were foreign-born or non-citizens (as defined per country or area), estimated incidence rates per 100,000 population (generated by WHO), and population size estimates [9]. A total of 36 countries and areas in the WHO WPR were analysed in this study, namely Australia, Brunei Darussalam, Cambodia, China, Hong Kong SAR (China), Japan, the Lao People’s Democratic Republic (Lao PDR), Macao SAR (China), Malaysia, Mongolia, New Zealand, Papua New Guinea, the Philippines, the Republic of Korea, Singapore, Viet Nam, and the Pacific Island countries and areas, including American Samoa (USA), Cook Islands, Fiji, French Polynesia, Guam, Kiribati, Marshall Islands, Micronesia (Federated States of), Nauru, New Caledonia, Niue, Northern Mariana Islands, Palau, Samoa, Solomon Islands, Tokelau, Tonga, Tuvalu, Vanuatu, and Wallis and Futuna. Pitcairn Island, which did not report TB data during the study period, and Indonesia, which was reassigned from the WHO South-East Asia Region to the WPR in 2025, were excluded.

Migrant data were extracted from the United Nations Department of Economic and Social Affairs’ (UN DESA) International Migrant Stock 2020 dataset [10]. For statistical purposes, UN DESA defines international migrants as individuals who have moved across an international border and changed their country of residence, irrespective of the reason for migration or legal status, including refugees. A threshold, usually 12 months, is used to specify the minimum period a person must have lived continuously in another country to qualify as a migrant. The dataset provides the estimated number (or “stock”) of international migrants by origin and destination for 232 countries and areas worldwide in five-year intervals [11]. In most countries, foreign-born individuals were counted as international migrants. However, where birth data were unavailable, foreign citizenship was used as a proxy [11]. This approach effectively equates foreign citizens with foreign-born migrants. Data used to estimate the migrant stock were primarily obtained or derived from national population censuses, supplemented by information from population registers and surveys. Interpolation and extrapolation were applied to estimate values for years without census data. Key indicators in this dataset included international migrant stock as a percentage of the total population (both sexes combined, by destination), and international migrant stock at mid-year (both sexes combined, by destination and origin). For origin countries lacking 2020 data, the most recent data prior to 2020 were used. These included American Samoa (2015), the British Virgin Islands (1995), the Northern Mariana Islands (1995), Lao PDR (2015), and Uruguay (2015).

### Foreign-born TB notification trends

At global and WHO regional levels, we examined the average annual number and proportion of foreign-born TB case notifications from 2021–2023, as well as trends in these proportions from 2019–2023. Across WPR countries and areas, we compared the average foreign-born TB proportions for 2021–2023 and trends in case notifications and proportions from 2008–2023. Proportions were calculated by dividing the number of foreign-born TB notifications by the total number of notified TB cases, expressed as a percentage. Global and regional data were aggregated from national-level data across the six WHO Regions: Africa, the Americas, South-East Asia, Europe, the Eastern Mediterranean, and the Western Pacific. Data from the 20 Pacific Island Countries in the WPR were also aggregated for cross-country analyses. Nine countries and areas with the highest number of foreign-born TB cases within the WPR were fitted with locally estimated scatterplot smoothing (LOESS) regression with a 95% confidence interval (CI) to facilitate the identification of underlying trends.

### Data reporting coverage

We assessed the TB data reporting coverage at global and regional levels by comparing the number of countries and areas that reported: 1) both overall and foreign-born TB cases, 2) only overall TB cases, and 3) neither.

### Estimated TB incidence among international migrants

To estimate TB incidence and TB incidence rate per 100,000 among international migrants in the WPR, we used data on: 1) the number of international migrants at each WPR destination, either aggregated (N_a_) or disaggregated by origin (N_o_), and 2) the TB incidence rate at each origin (IR) as generated by WHO [8, 10].

We estimated the TB incidence at each WPR destination by: 1) multiplying N_o_ by IR and dividing by 100,000 to determine incidence at each origin, and 2) summing the results. Regional incidence was estimated similarly by aggregating cases across all WPR destinations. For origins classified as “Others” in the International Migrant Stock Dataset, the global estimated incidence rate was applied in place of country-specific rates.

We then estimated the TB incidence rate per 100,000 at each WPR destination by dividing the estimated TB incidence among international migrants by N_a_, and multiplying by 100,000. The regional rate was determined similarly, using the total estimated TB incidence and the total number of international migrants in the WPR.

In a separate analysis, we calculated the proportion of TB incidence attributed to: 1) the 30 high TB burden countries (HBC) and 2) countries and areas outside the WPR. This was done by dividing the number of TB incidence cases among international migrants from each of these origins by the regional total, expressed as a percentage.

Additionally, we evaluated the association between the proportion of notified foreign-born TB cases and the proportion of international migrants in WPR destinations using Spearman’s correlation. Shapiro’s test was applied to assess normality.

The significance level was set at 0.05. All data analyses and visualisations were performed using the statistical software package R 4.4.2 (Comprehensive R Archive Network: https://cran.r-project.org/). Ethical approval was not required for this study as it used publicly available data with no individually identifiable information.

## Results

### Trends in foreign-born TB globally and across WHO Regions

**Fig. 1:**
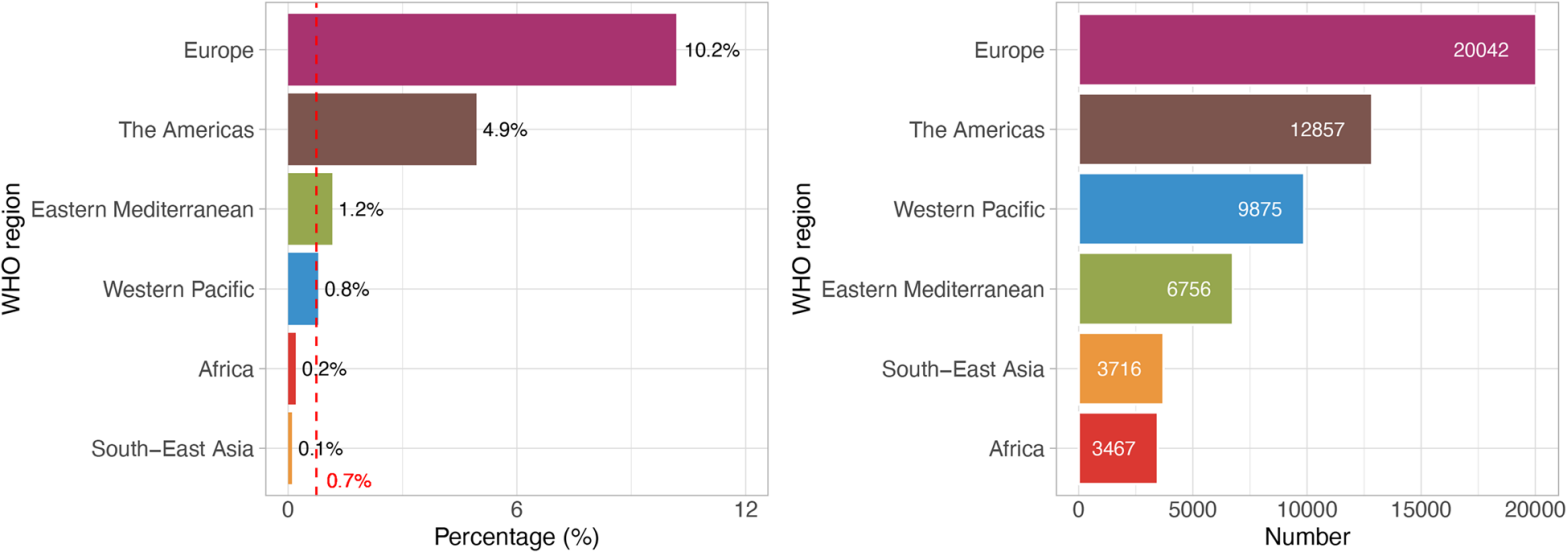
Proportion and average number of foreign-born tuberculosis (TB) cases by WHO Region, 2021–2023. The proportion of foreign-born cases among total TB notifications across the six WHO Regions (left), and the average annual number of foreign-born TB cases in these regions (right). The red dotted line indicates the global estimated level

In 2020, international migrants constituted an estimated 3.5% (275 million) of the global population [10]. From 2021–2023, an average of 56,714 TB cases among foreign-born individuals were reported worldwide, representing approximately 0.7% of total TB notifications (Fig. 1). Regional disparities were marked, with the WHO European Region reporting the highest proportion of foreign-born TB cases (20,042 cases annually; 10.2% of total regional notifications), followed by the Americas (12,857; 4.9%), the Western Pacific (9,875; 0.8%) and the Eastern Mediterranean (6,756; 1.2%). In comparison, the South-East Asia and Africa Regions recorded the lowest proportions, with 3,716 cases (0.1%) and 3,467 cases (0.2%) of foreign-born TB cases annually, respectively.

**Fig. 2:**
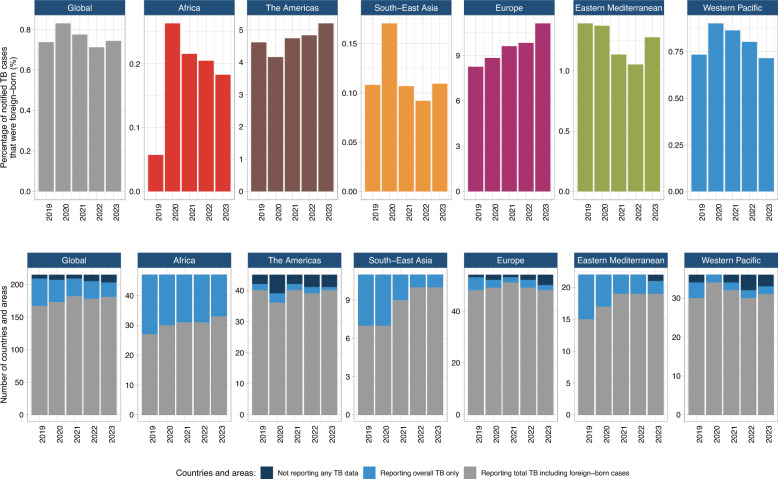
Proportions of foreign-born tuberculosis (TB) cases and TB reporting coverage globally and by WHO Regions, 2019–2023. Five-year trends in the proportion of notified TB cases that were foreign-born (top). Reporting coverage for foreign-born and overall TB notifications during the same period (bottom)

Over the five years from 2019–2023, the global proportion of foreign-born TB notifications remained relatively stable between 0.7 and 0.8% (Fig. 2, upper panel). However, upward trends were evident in regions with higher foreign-born TB burdens. The proportion of foreign-born TB cases in Europe increased from 8.3 to 11.1%, while the Americas rose from 4.6 to 5.2%. In the WPR, the proportion peaked at 0.9% in 2020 before declining to 0.7% by 2023. Similarly, Africa experienced a peak of 0.3% in 2020 before a subsequent reduction. In contrast, the proportions in the Eastern Mediterranean (1.1–1.4%) and South-East Asia (0.1–0.2%) showed minimal fluctuations.

The coverage of TB reporting and foreign-born TB data remained robust in many WHO Regions. Between 2019 and 2023, the number of countries and areas reporting overall TB notifications declined slightly, from 209 to 203 (Fig. 2, lower panel). Reporting coverage remained relatively stable in the Americas, Europe and the Western Pacific, where foreign-born TB notifications were consistently reported. Improvements in coverage were observed in the Africa, the South-East Asia and the Eastern Mediterranean Regions.

### Trends in foreign-born TB notifications in the WPR

**Fig. 3:**
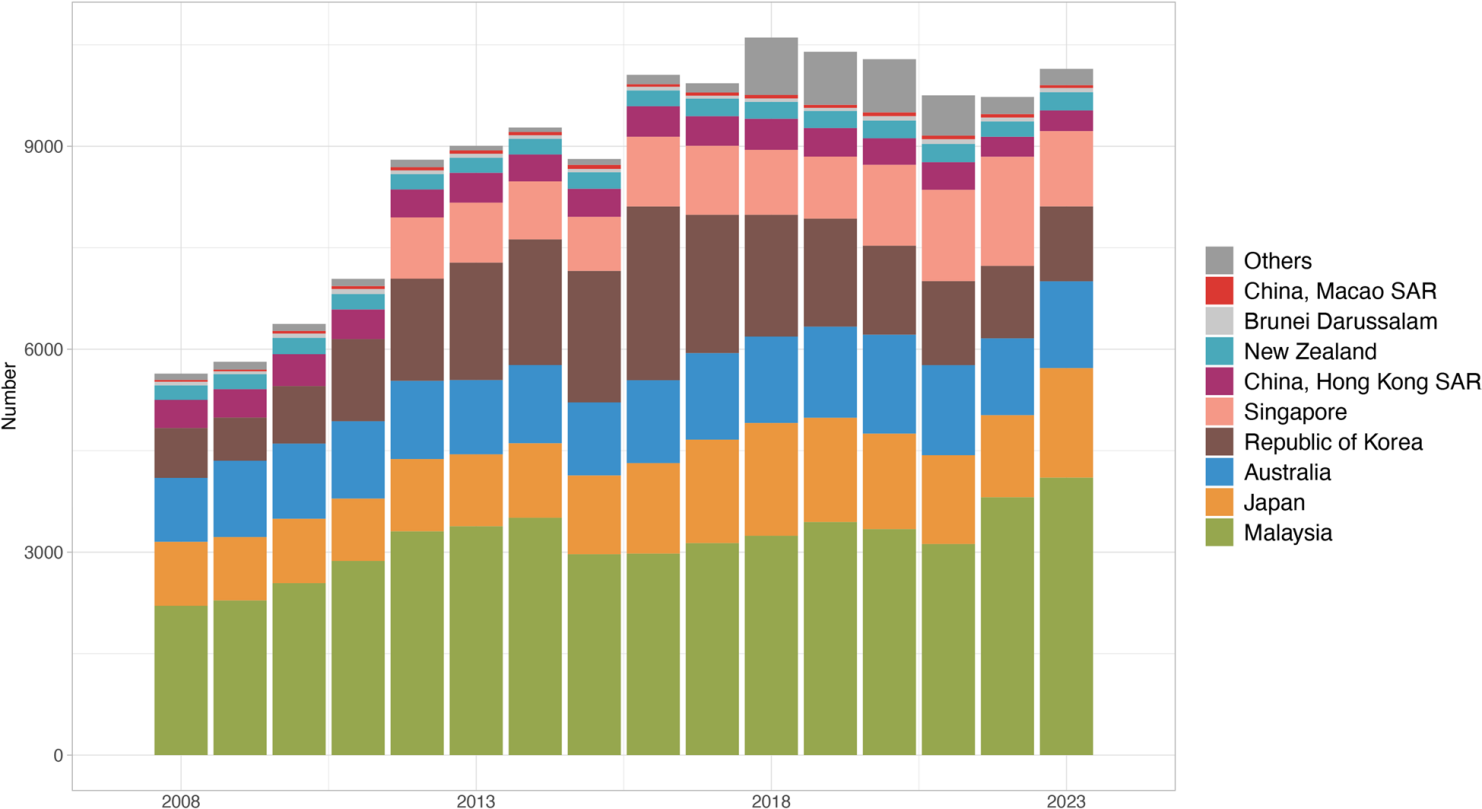
Number of notified foreign-born tuberculosis (TB) cases by destination country and area in the Western Pacific Region, 2008–2023. Annual trend in the number of foreign-born TB notifications over the 15-year period

Between 2008 and 2023, the number of foreign-born TB notifications in the WPR increased. Cases rose from 5,639 in 2008 to 10,056 in 2016, representing a 78.3% increase (Fig. 3). Since 2016, notifications have stabilised, fluctuating between 9,729 and 10,607 cases annually. In 2023, Malaysia accounted for the largest share of foreign-born TB notifications in the region (40.4%), followed by Japan (16.0%), Australia (12.7%), Singapore (11.0%), and the Republic of Korea (10.9%). Collectively, these five countries, along with Hong Kong SAR, New Zealand, Brunei Darussalam, and Macao SAR, accounted for 97.6% of the total number of foreign-born notifications in the region.

### Country-specific notification trends in the WPR

**Fig. 4:**
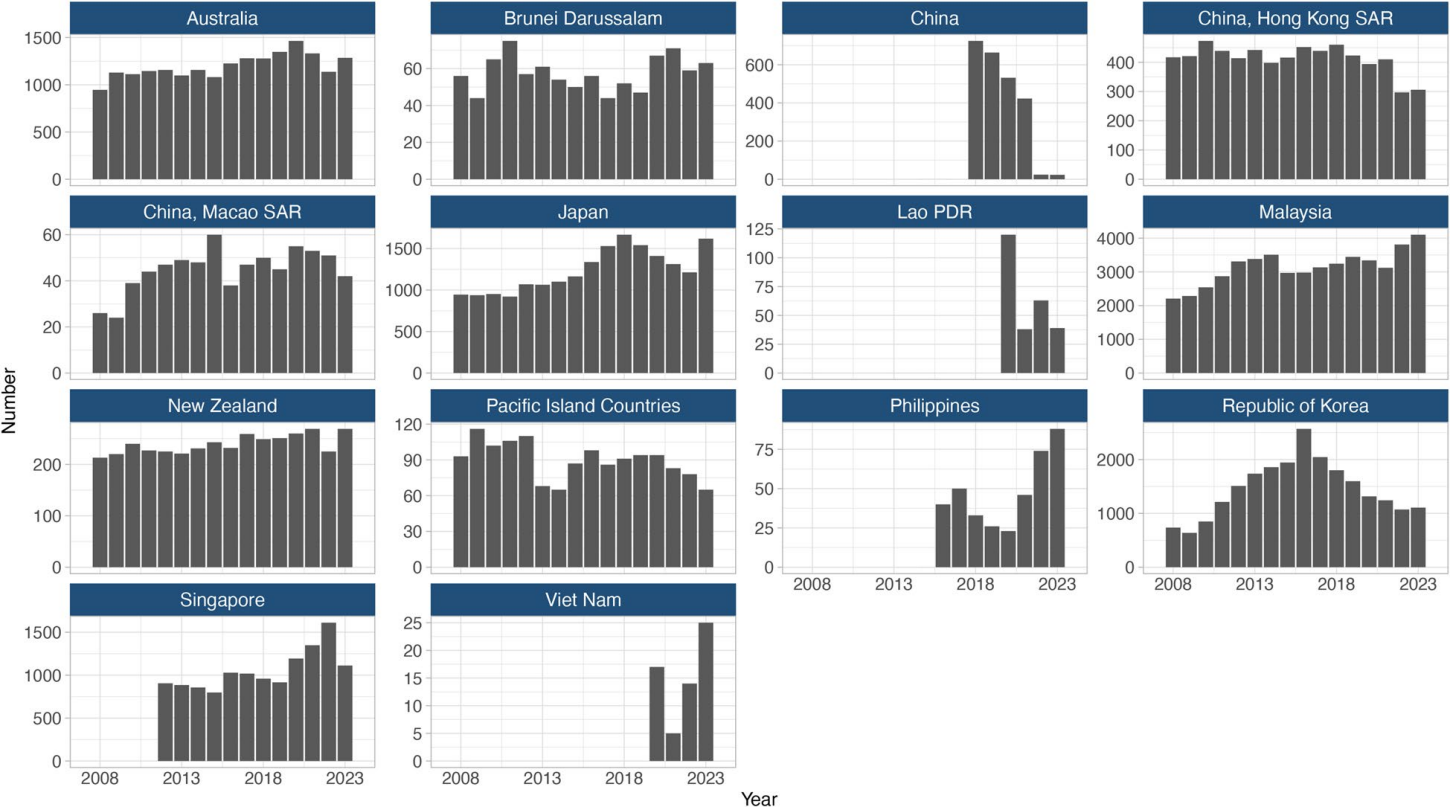
Number of notified foreign-born tuberculosis (TB) cases in fourteen selected destination countries and areas in the Western Pacific Region, 2008–2023. Annual trends in the number of TB notifications that were foreign-born over the 15-year period. Pacific Island Countries included American Samoa, Cook Islands, Fiji, French Polynesia, Guam, Kiribati, Marshall Islands, Micronesia (Federated States of), Nauru, New Caledonia, Niue, Northern Mariana Islands, Palau, Samoa, Solomon Islands, Tokelau, Tonga, Tuvalu, Vanuatu, and Wallis and Futuna Islands. China, Lao PDR, the Philippines, and Viet Nam began reporting foreign-born TB cases between the late 2010s and early 2020s. Singapore adopted the current case definitions in 2012 to include both long-staying foreigners and foreign-born residents

The trends in foreign-born TB notification varied across countries and areas in the WPR, reflecting differing migration patterns, healthcare systems, and TB control strategies (Fig. 4). Malaysia and Singapore demonstrated consistent increases, with Malaysia’s foreign-born TB cases rising from 2,207 in 2008 to 4,101 in 2023, and Singapore’s cases increasing from 905 in 2012 to 1,112 in 2023. In contrast, China and Lao PDR reported consistent declines. Cases decreased from 725 in 2018 to 23 in 2023 for China, and from 120 in 2020 to 39 in 2023 for Lao PDR.

The Republic of Korea and Japan experienced fluctuating trends. The Republic of Korea saw a peak of 2,569 cases in 2016, followed by a decline to 1,107 cases in 2023. Similarly, Japan’s foreign-born TB notifications peaked at 1,667 cases in 2018 before decreasing to 1,214 cases in 2022, then rising again to 1,619 cases in 2023. In contrast, Australia (946–1,464 cases), New Zealand (213–269 cases), Pacific Island Countries (65–116 cases), Brunei Darussalam (44–75 cases), and Macao SAR (24–60 cases) showed relatively stable trends. Hong Kong SAR reported a decline from 460 cases in 2018 to 306 in 2023. Conversely, TB notifications in the Philippines declined to a low of 23 cases in 2020 before increasing to 88 in 2023, while Viet Nam experienced a similar rebound, rising from 5 to 25 cases over the same period.

### Proportion of foreign-born TB cases within the WPR

**Fig. 5:**
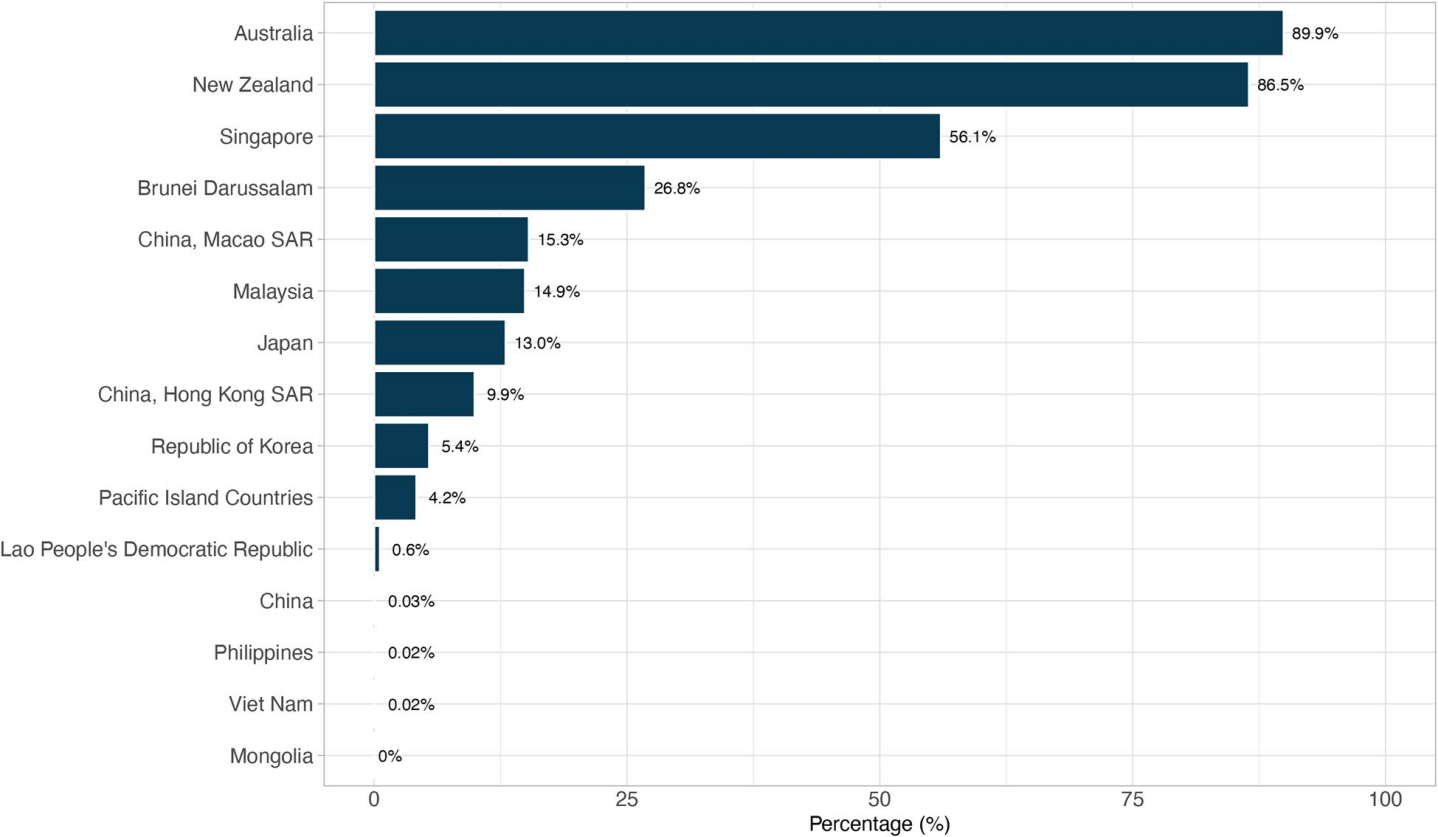
Proportion of notified foreign-born tuberculosis cases by country and area in the Western Pacific Region, 2021–2023. Pacific Island Countries included Cook Islands, Fiji, French Polynesia, Guam, Kiribati, Marshall Islands, Micronesia (Federated States of), Nauru, New Caledonia, Niue, Northern Mariana Islands, Palau, Samoa, Solomon Islands, Tokelau, Tonga, Tuvalu, and Vanuatu. American Samoa and Wallis and Futuna Islands did not report these data. Cambodia reported zero cases in 2022 but was excluded because data were not available for other years

The proportion of notified foreign-born TB cases relative to total TB cases provides a clearer understanding of the burden within specific countries and areas. Between 2021 and 2023, Australia (89.9%), New Zealand (86.5%) and Singapore (56.1%) reported the highest proportions of foreign-born TB cases (Fig. 5). Other countries and areas, including Brunei Darussalam, Macao SAR, Malaysia, Japan, and Hong Kong SAR, reported proportions ranging from 9.9% to 26.8%. Lao PDR, China, the Philippines, and Viet Nam reported proportions below 1.0%, while Mongolia reported no foreign-born TB cases.

**Fig. 6:**
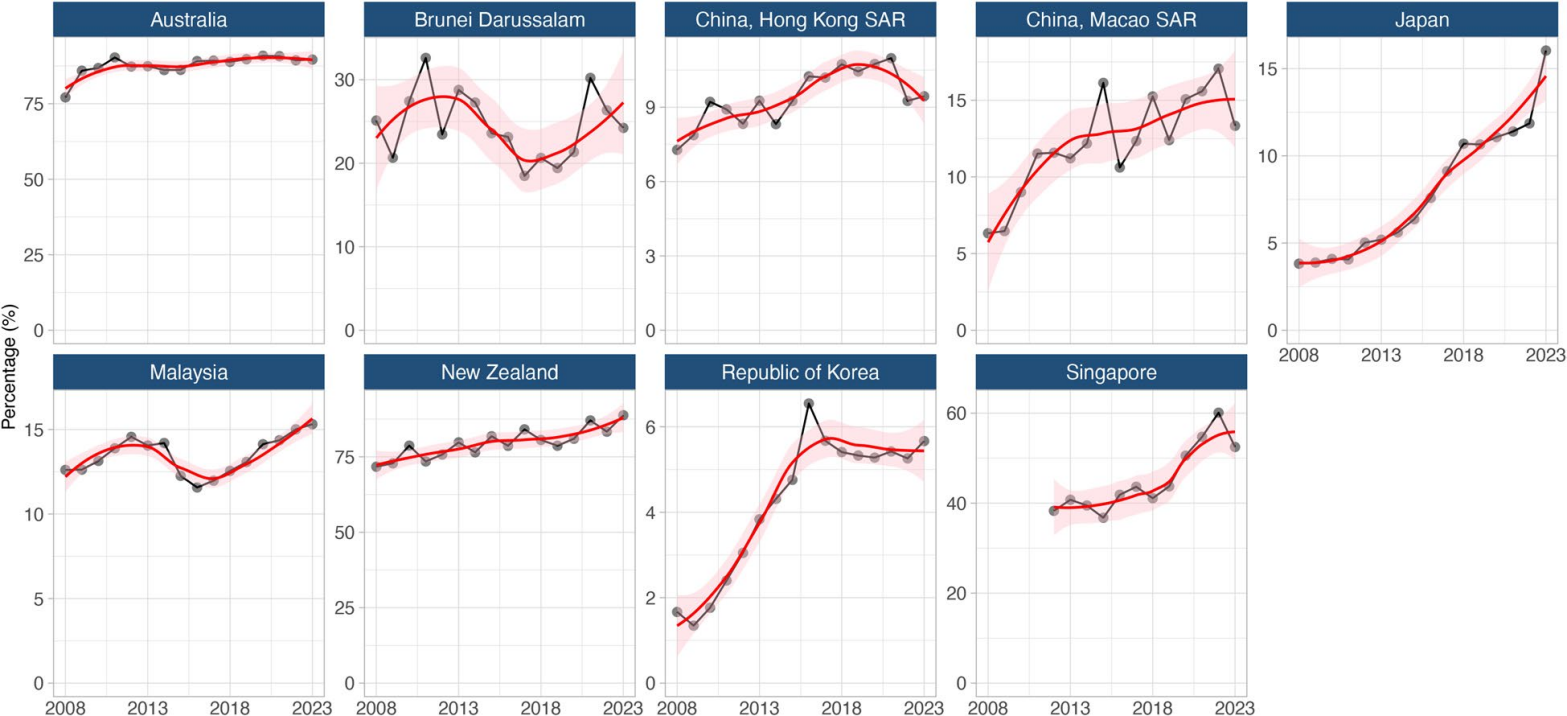
Proportion of notified foreign-born tuberculosis cases in nine selected destination countries and areas in the Western Pacific Region, 2008–2023. Regression lines (red) with 95% confidence intervals (pink shaded area) were fitted using the locally estimated scatterplot smoothing (LOESS) method

Several countries and areas showed a steady increase in foreign-born TB proportions over the 15-year period (2008–2023) (Fig. 6). Australia increased from 77.1% to 89.6%, New Zealand from 71.7% to 88.8%, Singapore from 38.3% to 52.5%, and Japan from 3.8% to 16.0%. In contrast, proportions in Brunei Darussalam (18.5–32.6%), Malaysia (11.6–15.3%), and Hong Kong SAR (7.3–11.0%) fluctuated within narrower ranges, while the Republic of Korea saw a spike to 6.5% in 2016, then stabilised between 5.3% and 5.7%.

### TB among international migrants in the WPR

**Fig. 7:**
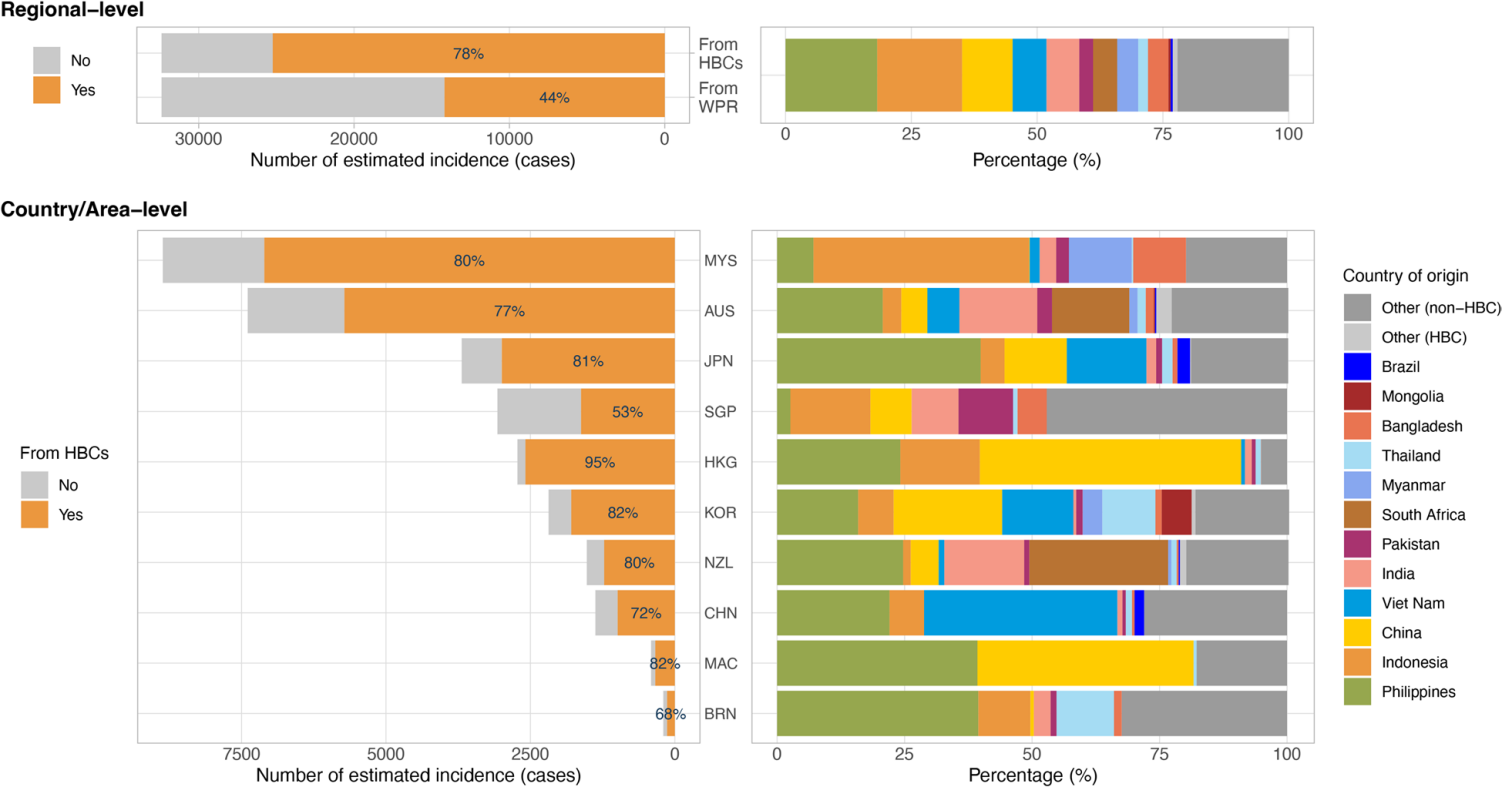
Sources of foreign-born tuberculosis (TB) cases in the Western Pacific Region (WPR). Top: Percentage of TB cases in the WPR originating from high-burden countries (HBCs) and from within the region (left), and composition of cases by country of origin (right). Bottom: Percentage of cases from HBCs (left), and composition of cases by country of origin among the ten countries and areas with the highest estimated number of cases (right). *MYS* Malaysia, *AUS* Australia, *JPN* Japan, *SGP* Singapore, *HKG* Hong Kong SAR (China), *KOR* the Republic of Korea, *NZL* New Zealand, *CHN* China, *MAC* Macao SAR (China), and *BRN* Brunei Darussalam

**Fig. 8:**
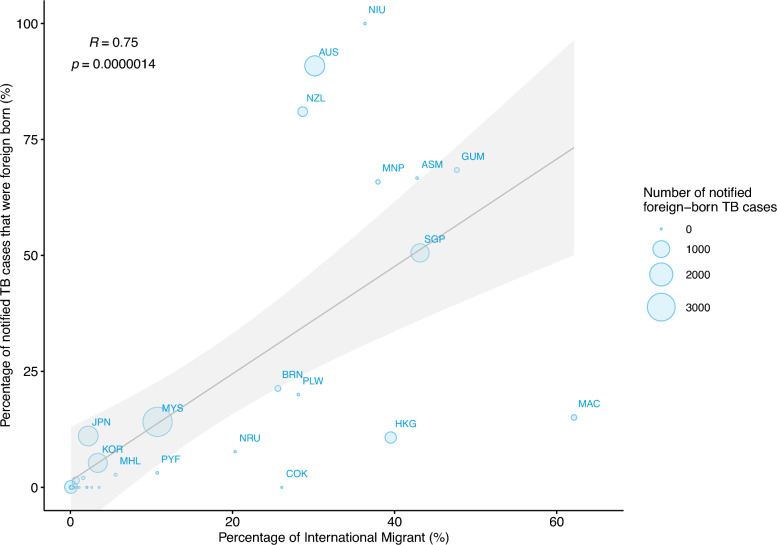
Association between the proportion of notified foreign-born tuberculosis (TB) cases and the proportion of international migrants in destination countries and areas in the Western Pacific Region, 2020. *ASM* American Samoa, *AUS* Australia, *BRN* Brunei Darussalam, *COK* Cook Islands, *GUM* Guam, *HKG* Hong Kong SAR (China), *JPN* Japan, *KOR* the Republic of Korea, *MAC* Macao SAR (China), *MHL* Marshall Islands, *MNP* Northern Mariana Islands (Commonwealth of the), *MYS* Malaysia, *NIU* Niue, *NRU* Nauru, *NZL* New Zealand, *PLW* Palau, *PYF* French Polynesia and *SGP* Singapore

In 2020, approximately 24.8 million international migrants resided in the WPR (Table 1), with 77.9% originating from high TB burden countries and 56.2% from outside the WPR (Fig. 7). Major source countries included the Philippines, Indonesia, China, Viet Nam, India, Pakistan, Myanmar, Thailand, and Bangladesh (Fig. 7). A statistically significant positive association was observed between the proportion of notified foreign-born TB cases and the proportion of international migrants in destination countries and areas (R = 0.75, p < 0.0001; Fig. 8).

**Table 1:** Population size, notified tuberculosis (TB) cases, and estimated TB incidence in the Western Pacific Region, by country and area and population type, 2020

Country/Area	Population			Notified TB cases			Estimated TB incidence (cases)			
	Total	International migrants (%)		Total	Foreign-born (%)		Destination country		Foreign-born population	
Australia	25,743,794	7,685,986	(29.9)	1,611	1,464	(90.9)	1,900	(1,600–2,100)	7,394	(5,351–9,865)
New Zealand	5,069,891	1,381,724	(27.3)	321	260	(81)	360	(310–420)	1,524	(1,114–2,020)
Singapore	5,620,149	2,523,648	(44.9)	2,362	1,194	(50.6)	2,700	(2,300–3,100)	3,071	(2,592–3,617)
Brunei Darussalam	447,400	111,959	(25)	314	67	(21.3)	360	(310–420)	199	(142–270)
China, Macao SAR	683,062	403,490	(59.1)	365	55	(15.1)	420	(360–480)	411	(310–536)
Malaysia	33,889,557	3,476,560	(10.3)	23,644	3,340	(14.1)	31,000	(26,000–35,000)	8,863	(6,835–11,282)
Japan	126,304,549	2,770,996	(2.2)	12,739	1,411	(11.1)	15,000	(13,000–17,000)	3,689	(2,619–5,004)
China, Hong Kong SAR	7,490,233	2,962,492	(39.6)	3,667	394	(10.7)	4,200	(3,600–4,800)	2,724	(2,156–3,412)
Pacific Island Countries	3,449,520	282,001	(8.2)	1,756	94	(5.4)	2,232	(1,737–2,802)	358	(230–521)
Republic of Korea	51,858,488	1,728,182	(3.3)	24,931	1,316	(5.3)	25,000	(23,000–26,000)	2,185	(1,556–2,958)
Lao PDR	7,346,525	48,731	(0.7)	8,122	120	(1.5)	11,000	(7,100–16,000)	73	(50–99)
China	1,426,106,090	1,039,675	(0.1)	633,156	532	(0.1)	834,000	(719,000–957,000)	1,375	(988–1,837)
Cambodia	16,725,472	79,341	(0.5)	29,139	0	(0)	47,000	(30,000–67,000)	122	(85–167)
Mongolia	3,290,785	21,347	(0.6)	4,091	0	(0)	14,000	(7,000–23,000)	16	(13–19)
Philippines	112,081,263	225,525	(0.2)	263,300	23	(0)	600,000	(336,000–941,000)	191	(169–216)
Papua New Guinea	9,815,741	31,068	(0.3)	29,959	0	(0)	42,000	(34,000–51,000)	52	(44–61)
Viet Nam	98,079,187	76,767	(0.1)	101,705	17	(0)	169,000	(107,000–241,000)	143	(108–184)
Western Pacific Region	1,934,001,706	24,849,492	(1.3)	1,141,182	10,287	(0.9)	1,800,000	(1,480,000–2,150,000)	32,390	(24,362–42,068)

Countries and areas in this table are sequenced according to the percentage of foreign-born TB cases notified. Data on population and notified TB cases were obtained from the WHO Global TB database. Estimated TB incidence was calculated using the data from the WHO Global TB database and the International Migrant Stock 2020 dataset (UN DESA). *Lao PDR* The Lao People's Democratic Republic, and *WHO* World Health Organization

**Fig. 9:**
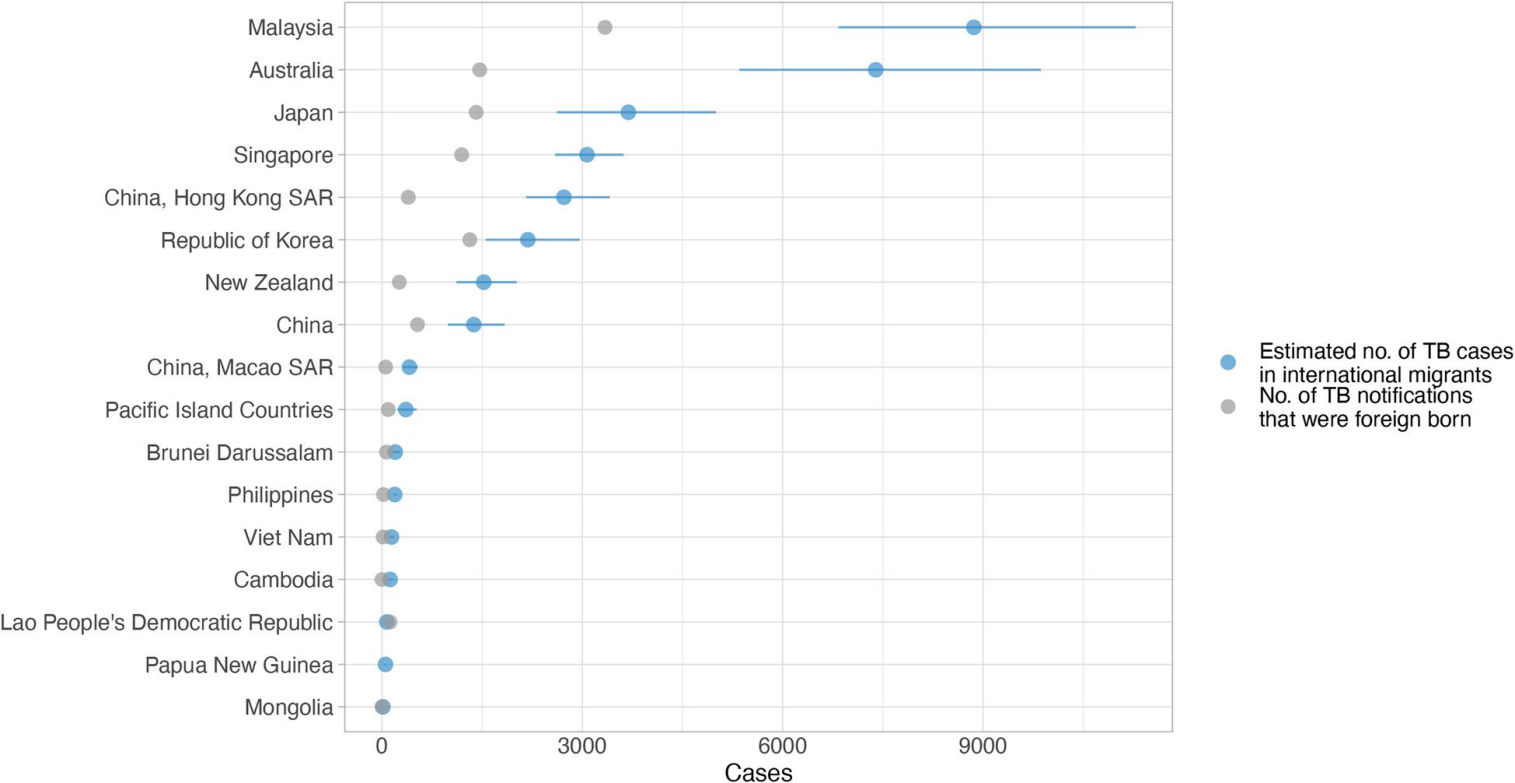
Estimated tuberculosis (TB) incidence in international migrants and number of notified foreign-born TB cases by destination country and area in the Western Pacific Region, 2020. Pacific Island Countries included American Samoa, Cook Islands, Fiji, Micronesia (Federated States of), Guam, Kiribati, Marshall Islands, Northern Mariana Islands, Niue, Nauru, Palau, French Polynesia, Solomon Islands, Tokelau, Tonga, Tuvalu, Vanuatu, Wallis and Futuna, and Samoa. Cambodia and Mongolia reported zero foreign-born TB cases. Data on notified cases that were foreign-born were not available for New Caledonia and Papua New Guinea in 2020

In this study, the TB incidence among international migrants in the WPR was estimated to be 32,390 cases (95% CI 24,362–42,068) in 2020. Malaysia (8,863 cases, 95% CI 6,835–11,282) and Australia (7,394 cases, 95% CI 5,351–9,865) had the highest estimated incidence, followed by Japan (3,689 cases, 95% CI 2,619–5,004), Singapore (3,071 cases, 95% CI 2,592–3,617), Hong Kong SAR (2,724 cases, 95% CI 2,156–3,412), and the Republic of Korea (2,185 cases, 95% CI 1,556–2,958) (Fig. 9). In several countries and areas, the estimated incidence among migrants exceeded the number of notified foreign-born TB cases, particularly in Malaysia (3,340 cases) and Australia (1,264 cases).

### Incidence rate comparison of international and domestic TB cases in WPR destination countries and areas

**Fig. 10:**
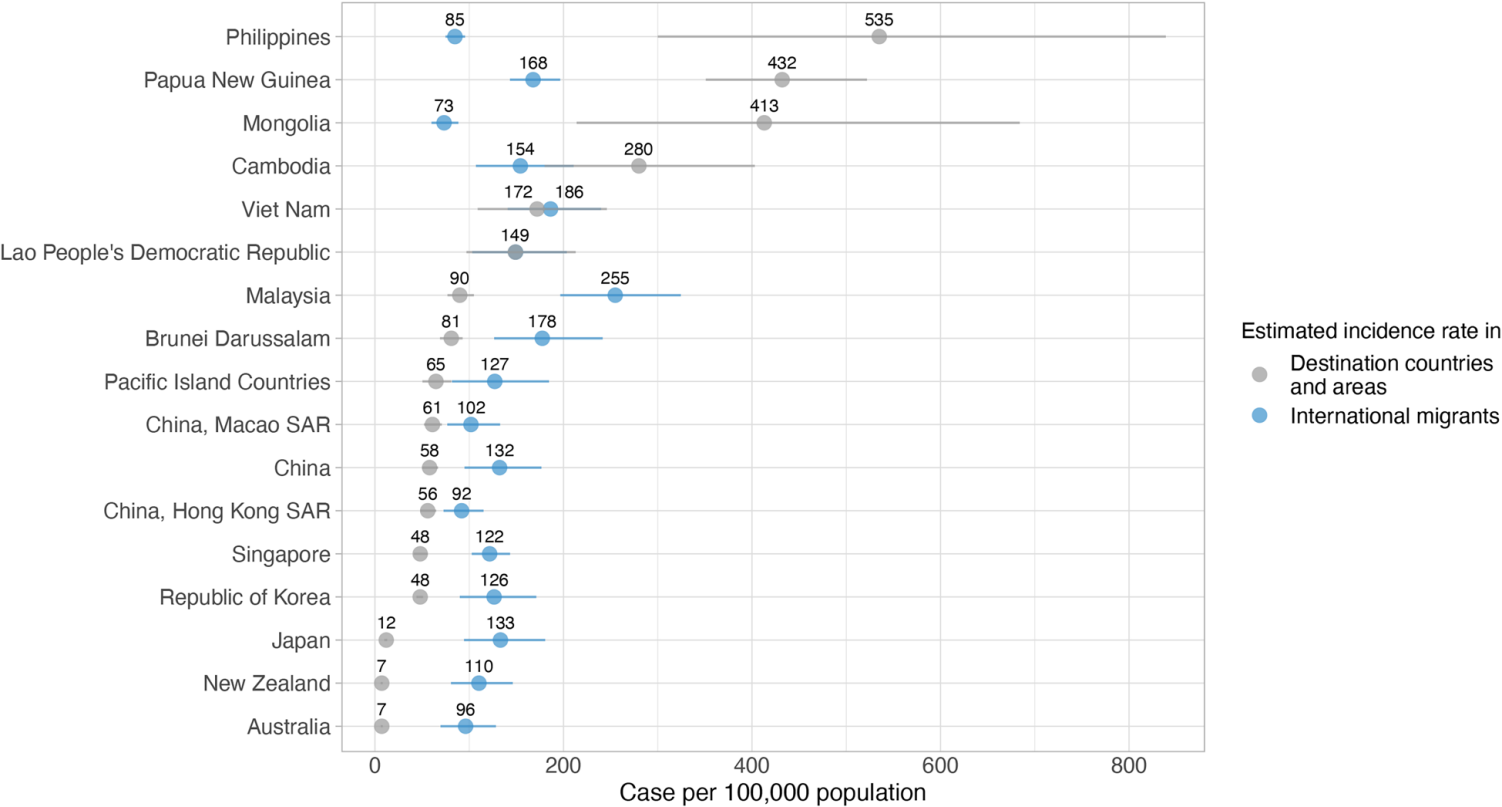
Estimated tuberculosis (TB) incidence per 100,000 population in international migrants and in destination countries and areas within the Western Pacific Region, 2020. Pacific Island Countries included American Samoa, Cook Islands, Fiji, Micronesia (Federated States of), Guam, Kiribati, Marshall Islands, Northern Mariana Islands, New Caledonia, Niue, Nauru, Palau, French Polynesia, Solomon Islands, Tokelau, Tonga, Tuvalu, Vanuatu, Wallis and Futuna, and Samoa

The estimated TB incidence rate among international migrants in the WPR in 2020 was 130 cases per 100,000 population (95% CI 98–169), often exceeding the national TB incidence rates in several WPR destinations (Fig. 10). Malaysia had the highest estimated migrant-specific incidence rate of 255 per 100,000, nearly 2.5 times the national rate of 90 per 100,000. Similarly, in Brunei Darussalam, the incidence among migrants was 178 per 100,000, compared with the national rate of 81 per 100,000. In Japan, the disparity was more pronounced, with migrants experiencing a TB incidence rate of 133 per 100,000, much higher than the national rate of 12 per 100,000. The Republic of Korea and Singapore also showed notable differences, with migrant incidence rates of 126 per 100,000 and 122 per 100,000, respectively, compared to the national average of 48 per 100,000. New Zealand and Australia followed similar patterns, with migrant rates of 110 per 100,000 and 96 per 100,000, respectively, far surpassing their domestic rates of 7 per 100,000.

Conversely, in certain countries with high TB burdens, national incidence rates exceeded those among international migrants. For example, the Philippines reported a national incidence rate of 535 per 100,000, significantly higher than the migrant rate of 85 per 100,000. Similarly, Papua New Guinea’s national incidence of 432 per 100,000 was well above the migrant-specific rate of 168 per 100,000. In Viet Nam and Lao PDR, TB incidence rates among migrants were comparable to national rates, reflecting relatively uniform TB dynamics.

## Discussion

Migration plays an important role in shaping TB epidemiology in the WPR and presents ongoing challenges to its control and elimination. This study provides the first region-wide analysis of the TB burden among foreign-born individuals, highlighting key trends and their implications for TB control in the WPR. Our findings indicate a substantial rise in regional foreign-born TB notifications between 2008 and 2016, followed by relative stability through 2023, with notable variations across countries and areas. The estimated TB incidence rate among international migrants (130 per 100,000 population) often exceeded national averages. These findings underscore the relevance of regionally coordinated and migrant-sensitive approaches for TB control in the WPR.

International migrants represent approximately 1.3% of the population in the WPR [2]. Foreign-born TB notifications accounted for 0.8% of total regional TB notifications, a lower proportion than in Europe (10.3%) and the Americas (4.9%), which host the largest international migrant populations [1]. Between 2008 and 2016, notifications in the WPR increased substantially before stabilising at around 10,000 cases annually from 2016 to 2023. Trends varied across the region, with some countries and areas reporting consistent increases (e.g. Malaysia and Singapore), while others showed fluctuations (e.g. the Republic of Korea and Japan) or declines (e.g. China and Lao PDR). These variations may reflect differences in migrant dynamics, economic development, and healthcare policies [5, 12]. The COVID-19 pandemic further affected TB notifications and migration dynamics [13, 14]. Regional foreign-born TB notifications reached their lowest levels since the start of the pandemic in 2021 and 2022, coinciding with widespread travel restrictions (e.g. screening, quarantining or banning arrivals, and total border closures) [2]. With measures relaxing through 2021, and largely lifted by 2022 or 2023, international migrant flows recovered and in some settings surpassed pre-pandemic levels [14, 15]. Country-level differences in notification trends are likely linked to variations in health and surveillance system resilience, socioeconomic conditions, border and visa policies, and the composition of migrant populations within destinations [13, 15, 16].

As of 2020, approximately 24.8 million international migrants resided in the WPR, representing 8.9% of the global international migrant population [10]. Among them, 56.2% came from outside the region and 77.9% originated from HBCs, including the Philippines, Indonesia, China, Viet Nam, India, Pakistan, Myanmar, Thailand, and Bangladesh. Consistent with previous studies, our findings show a strong positive correlation between the proportion of foreign-born TB cases and the proportion of international migrants in the population, particularly in settings with a large share of migrants from HBCs [17, 18]. Regionally, the estimated TB incidence rate among international migrants (130 per 100,000 overall and 73–254 per 100,000 in low-to-intermediate burden settings) aligns with the range reported in a recent systematic review of migrants, refugees, and asylum seekers across different contexts (19–754 per 100,000) [19]. The discrepancy between the estimated TB incidence among international migrants (32,390 cases) and the number of foreign-born TB notifications (10,287 cases) in the present study may be related to the implementation of pre-entry TB screening programmes, differences in migrant profiles, and underdetection or underreporting of cases [5, 20].

Migrant TB screening policies and migration dynamics significantly influence foreign-born TB notifications and may help to explain the observed trends and disparities [5, 21]. High-income countries with robust screening policies and surveillance systems often report substantial proportions of foreign-born cases relative to their total notifications (e.g. > 86% in Australia and New Zealand) [22–25]. In line with previous reports, our study showed that the estimated TB incidence rates among international migrants were markedly higher than those in the general population in these countries (14-fold in Australia, 96 vs. 7 per 100,000; 16-fold in New Zealand, 110 vs. 7 per 100,000) [22, 25]. This may reflect the role of post-migration and follow-up screening measures  in enhancing case finding and supporting earlier detection and reporting of foreign-born TB cases [26–28]. These findings underscore the relevance of targeted screening in low-incidence settings, where the TB burden is largely driven by foreign-born cases. 

Screening policies also shape foreign-born TB trends in intermediate-burden countries, some of which are major migrant destinations and transit hubs in the region. Our results suggest that the TB incidence rate among international migrants can be 1.6–2.8 times higher than in the general population in these settings. In the Republic of Korea, the introduction of mandatory TB screening for long-term visa applicants in 2016 initially led to a peak in foreign-born TB cases, followed by declines as early detection and prevention strategies took effect [29, 30]. Similarly, the upward trend in foreign-born TB notifications in Singapore, linked to expanded surveillance policies in 2012, highlights the influence of policy changes [31]. In contrast, Malaysia faces challenges in implementing uniform screening programmes and enforcing TB notification, potentially contributing to the notable gap between the estimated TB incidence rate among migrants (282 per 100,000) and the national rate (140 per 100,000) [32–34].

Conversely, high-burden migrant-origin countries that contribute significantly to regional and global migration flows, such as the Philippines and Viet Nam, often report very low proportions of foreign-born TB (< 1%). TB incidence among international migrants is substantially lower than national rates in some settings, for example, in the Philippines (85 vs 525 per 100,000), Papua New Guinea (167 vs 432 per 100,000), and Mongolia (73 vs 413 per 100,000). These discrepancies may arise from lower inflows of international migrants from other HBCs, and the exclusion of undocumented or irregular migrants, often from neighbouring countries and areas, from official statistics [7, 35–37]. Suboptimal surveillance systems, underreporting, and limited resources for cross-border health initiatives further contribute to challenges in these contexts [6, 35, 38].

Migration-related TB challenges in the WPR are both shared and regionally specific. Common issues include limited healthcare access, delayed diagnoses, and socioeconomic vulnerabilities such as poverty, overcrowding and precarious employment [6, 7]. The underrepresentation of vulnerable groups, including undocumented migrants, refugees, and mobile populations, remains a concern [5, 6, 39]. This is particularly evident in cross-border regions and along archipelagic and remote borders, such as the Greater Mekong subregion, the Philippines and Papua New Guinea [38]. The WPR’s unique position as both a source and destination for migrants adds further complexity. For instance, HBCs such as the Philippines and China face the dual burden of managing domestic TB while addressing migration-related risks, whereas migrant-receiving countries contend with higher TB incidence among foreign-born populations [4, 40]. Geographical and economic disparities also complicate TB control. Pacific Island Countries, despite reporting low foreign-born TB cases, face resource constraints that limit effective surveillance and care delivery [7, 41]. A multipronged approach tailored to the WPR context may help address these challenges. Expanding pre-migration and post-migration TB screening programmes for high-risk populations may support earlier detection and reduce transmission [26, 28]. Harmonised policies and enhanced cross-border coordination could improve care continuity, particularly for transient or seasonal migrants [38, 42]. Establishing bilateral or multilateral TB referral mechanisms could facilitate more efficient information exchange and strengthen cross-border tracking and follow-up [43–46]. Public–private partnerships and community-based interventions may extend healthcare access to undocumented and vulnerable populations. Tackling broader social determinants, including housing and nutrition, and comorbidities such as diabetes and HIV, remains essential [6, 42]. Regional collaboration in monitoring foreign-born TB trends and improving international patient referral could further strengthen surveillance, optimise targeted interventions, and streamline resource allocation [40, 47, 48].

This study has several limitations. Firstly, the number and proportion of foreign-born TB cases may be underestimated due to incomplete reporting coverage across WHO Regions and within the WPR, compounded by COVID-19-related disruptions [13]. The exclusion of populations such as irregular and undocumented migrants may further underestimate the true incidence of foreign-born TB [40, 48]. These groups constitute a hidden epidemic, contributing to underreporting and potentially sustaining transmission by remaining undiagnosed and untreated. In addition, inconsistencies in defining “foreign-born” (e.g. by place of birth or country of citizenship) may hinder comparability, and the binary definition may omit or underrepresent some migrant subgroups, including those with irregular status or dual citizenship. Secondly, international migrant stock may be underestimated as short- and medium-term visitors or migrants are not included in the standard definition of “international migrant”. Differences in national definitions (e.g. the minimum duration of residence or the use of place of birth vs. citizenship) and variations in data collection methodologies further complicate interpretation [49]. Thirdly, estimating TB incidence among international migrants using incidence rates in their countries of origin may not fully capture the complexity of the situation, as migrant subpopulations vary in risk and composition across countries [50]. Despite these limitations, this study provides the first regional analysis of foreign-born TB in the WPR using harmonised and publicly available global datasets, enabling the estimation of TB burden among international migrants. These findings establish a critical baseline for regional comparisons and highlight opportunities for strengthening TB control among migrant populations in the WPR.

To strengthen the evidence base on foreign-born TB, future work should prioritise enhancing routine and continuous migration and TB surveillance to improve data completeness and allow more robust trend monitoring over time. Greater harmonisation of definitions used in TB and migrant surveillance would further enhance comparability across countries and regions. Integrating innovative data sources that capture different aspects of international migration, such as social big data and Displacement Tracking Matrix data from the International Organization for Migration, may help fill information gaps, refine estimates, and provide more timely and granular data [51–53]. In addition, incorporating migration-related factors, such as duration of residence, migration pathway, and key demographic characteristics, may enable more detailed analyses of risk and vulnerability, and support the development of targeted, evidence-informed interventions [52].

## Conclusions

To our knowledge, this study provides the first regional analysis of foreign-born TB burden in the WPR. It identifies substantial variations in notifications across the region, and differences between the estimated incidence among international migrants and national averages. Strengthening TB and migration surveillance, improving data comparability, and promoting cross-border collaboration could help address existing gaps and support more coordinated responses. The findings also highlight areas where further evidence and migrant-sensitive approaches may support more equitable progress towards the End TB goals.

## Data Availability

The data that support the findings of this study are available from the WHO Global Tuberculosis Database, https://www.who.int/teams/global-tuberculosis-programme/data, and the United Nations Department of Economic and Social Affairs’ (UN DESA) International Migrant Stock dataset, https://www.un.org/development/desa/pd/content/international-migrant-stock

## Abbreviations

HBC: High tuberculosis burden countries
IR: Tuberculosis incidence rate at each origin
LOESS: Locally estimated scatterplot smoothing regression
N_a_: The number of international migrants at each Western Pacific Region destination, aggregated
N_o_: The number of international migrants at each Western Pacific Region destination, disaggregated by origin
TB: Tuberculosis
UN DESA: United Nations Department of Economic and Social Affairs
WPR: Western Pacific Region
WHO: World Health Organization
